# Highly efficient surface-emitting semiconductor lasers exploiting quasi-crystalline distributed feedback photonic patterns

**DOI:** 10.1038/s41377-020-0294-z

**Published:** 2020-04-09

**Authors:** Simone Biasco, Andrea Ciavatti, Lianhe Li, A. Giles Davies, Edmund H. Linfield, Harvey Beere, David Ritchie, Miriam S. Vitiello

**Affiliations:** 1 NEST, CNR - Istituto Nanoscienze and Scuola Normale Superiore, Piazza San Silvestro 12, 56127 Pisa, Italy; 2 School of Electronic and Electrical Engineering, University of Leeds, Leeds, LS2 9JT UK; 3 Cavendish Laboratory, University of Cambridge, Cambridge, CB3 0HE UK

**Keywords:** Quantum cascade lasers, Electronics, photonics and device physics

## Abstract

Quasi-crystal distributed feedback lasers do not require any form of mirror cavity to amplify and extract radiation. Once implemented on the top surface of a semiconductor laser, a quasi-crystal pattern can be used to tune both the radiation feedback and the extraction of highly radiative and high-quality-factor optical modes that do not have a defined symmetric or anti-symmetric nature. Therefore, this methodology offers the possibility to achieve efficient emission, combined with tailored spectra and controlled beam divergence. Here, we apply this concept to a one-dimensional quantum cascade wire laser. By lithographically patterning a series of air slits with different widths, following the Octonacci sequence, on the top metal layer of a double-metal quantum cascade laser operating at THz frequencies, we can vary the emission from single-frequency-mode to multimode over a 530-GHz bandwidth, achieving a maximum peak optical power of 240 mW (190 mW) in multimode (single-frequency-mode) lasers, with record slope efficiencies for multimode surface-emitting disordered THz lasers up to ≈570 mW/A at 78 K and ≈720 mW/A at 20 K and wall-plug efficiencies of *η* ≈ 1%.

## Photonics: Unusual patterns put the shine on wire-like lasers

Researchers report that ‘quasicrystal’ patterns — mathematical sequences that are ordered, but never repeat themselves — can help turn tiny wires into high-efficiency lasers. Microchip-scale photonic lasers are normally produced by etching semiconductor surfaces with periodic arrays of optical microcavities. Miriam Serena Vitiello from CNR-Istituto Nanoscienze in Pisa, Italy, and colleagues now show that arranging microcavities into quasicrystal patterns can make it easier to pack more photonic lasers onto computer chips. The team first carved grating-like wire structures into the surface of gallium arsenide-based materials, then lithographically etched a series of non-repeating microcavities into each wire. Tuning the widths of the wires and the dimensions of the cavities enabled the devices to exhibit both single-frequency and broadband terahertz laser emissions, while providing improved power characteristics compared to conventional patterning methods.

## Introduction

The photonic engineering of semiconductor laser cavities has been extensively investigated in recent years as a versatile approach to control the spectral, spatial and temporal emission of lasers operating in different regions of the electromagnetic spectrum^1,2^.

Patterned resonators can be exploited to create miniaturised and efficient laser sources by using one-dimensional (1D)^3^ or two-dimensional (2D)^4–6^ patterning to modulate the optical properties of a cavity spatially, hence directly controlling the feedback and extraction mechanisms. The patterns can be either periodic or aperiodic. Periodic patterns exploit fully ordered perturbation elements (such as slits, holes or groves) that induce a well-defined photonic band structure. In this case, light propagation is forbidden within the frequency bandgaps, and laser operation is typically achieved at frequencies corresponding to the photonic band edges^7^. In contrast, aperiodic structures are characterised by an irregular distribution of scattering elements, which can be either randomly defined or designed using a deterministic generation rule, leading to optical modes that are critically localised inside the resonator^8^.

Periodic patterning has been successfully exploited in combination with miniaturized quantum cascade laser (QCL) semiconductor heterostructures operating at mid-IR^9^ and terahertz (THz) frequencies^10^, where unconventional resonator architectures have been used to tailor the emission frequency^11–13^ optical power^14^ beam divergence^14–17^ and direction^18^. This approach has been exploited to target relevant technological applications in spectroscopy, imaging, sensing and metrology^19–21^. 1D and 2D regular photonic structures have been applied to micro-ring mid-IR and micro-disk THz lasers^22,23^, photonic crystal lasers^24–26^, and distributed feedback (DFB) lasers^10,12–15,27^, demonstrating the robustness of this approach across different frequency ranges and laser architectures^28,29^.

Periodic resonators typically rely on electromagnetic eigenmodes with well-defined symmetric and anti-symmetric spatial distributions. In general, anti-symmetric modes display destructive interference in the far-field, have high quality factors and are strongly confined in the cavity. Symmetric modes, in contrast, constructively interfere in the far-field and produce more efficient light extraction into free space at the expense of low quality factors. Therefore, in comparison with symmetric eigenmodes, anti-symmetric modes are more likely to lase, but the emitted power will be low, except in the case of short cavity devices with chirped^30^ or π-shifted gratings^31^, which can allow efficient anti-symmetric mode emission. The competition between symmetric and anti-symmetric modes usually leads to a non-negligible power cancellation in the far-field.

This problem can be addressed, however, by devising architectures in which the resonator periodicity is deliberately perturbed by including localised defects^13^ or by implementing hybrid DFB patterns, exploiting a combination of second- and fourth-order gratings that are chosen to maximise the surface-emitted power (up to 170 mW) and slope efficiency (up to 993 mW/A) for single-spatial-mode lasers^32^. The first approach has been demonstrated in dual periodicity gratings^18^, laterally corrugated wire lasers with a surface extraction grating^28^, graded photonic heterostructures (peak powers of 100 mW and slope efficiencies 230 mW/A)^33,34^, and non-deterministic disordered structures such as random THz lasers with intrinsically multimode emission both in 1D^35^ and 2D configurations^36–39^ (see the performances in Table 1) and a unique low spatial coherence of the emitted optical modes.

**Table 1:** Performance comparison

	Peak power	Slope efficiency	Single mode/multi mode
3rd order DFB	11 mW	142 mW/A	Single mode
1D corrugated DFB	25 mW	250 mW/A	Single mode
2D–7-fold quasi crystal^a^	67 mW	57 mW/A	Multi mode
2D random laser^a^	21 mW	18 mW/A	Multi mode
Octonacci quasi crystal laser	190 mW	720 ± 16 mW/A 570 ± 14 mW/A (78 K)	Single mode
Octonacci quasi crystal laser^a^	240 mW	700 ± 12 mW/A 570 ± 11 mW/A (78 K)	Multi mode

Comparison between the peak output power and slope efficiency of a set of 1D and 2D single-frequency-mode and multimode THz lasers fabricated from the same heterostructure

^a^Indicates devices with identical dimensions

Lying in-between perfectly periodic structures and completely disordered structures, photonic quasi-crystal resonators^40^ exploit discrete translational invariance generated by a deterministic algorithm and feature long-range order^41,42^ that does not allow quasi-crystal resonators to be described with a classical band structure picture. Quasi-crystals can support extended band-like states at the edges of their spectral pseudo-gaps like periodic photonic crystals but can also support localised modes analogous to Anderson modes in completely random structures^8^. The single continuous spectrum of a quasi-crystal^40^ can be easily engineered to control the energy spacing and the position of the mode independently, allowing more than one Bragg resonance to be exploited for feedback or allowing separate Bragg resonances for feedback and out-coupling, which can lead to controlled collimated emission at a desired angle, without affecting the lasing frequency. In turn, this approach determines a complex light interference pattern, typically selecting a number of resonating (radiative) eigenmodes with large quality factors.

To date, quasi-crystal lasers have been exploited in combination with THz QCL heterostructures to tailor the emission spectra, control the light feedback and outcoupling, and shape the emitted beams, exploiting either one-dimensional architectures such as Fibonacci gratings^43^ or two-dimensional geometries such as a five-fold Penrose pattern^11^ or a seven-fold pattern with different rotational symmetries^44^.

1D geometries combine the typical advantages of quasi-periodic structures (directional output independent of the emission frequency and multicolour operation) with the potential to achieve good thermal management, enabled by the compact device size, which is beneficial for practical applications.

However, to date, laser emission has been limited in such architectures, with output peak power levels <6 mW and negligible wall-plug efficiencies (~0.01%)^43^ that have hampered the use of this technology for applications in chemical and biomolecular sensing and spectroscopy, where high-power, monolithic single-mode THz QCLs are necessary. A summary of the performances of a selection of aperiodic or disordered photonic structures is reported in the supplementary information file (Table S1).

Here, we conceive and demonstrate quasi-crystal THz QCL resonators that exploit a surface grating following the Octonacci design and are capable of significantly boosting the state-of-the-art performance of surface-emitting THz lasers. By tuning the laser width and the patterning slit size, the interplay between the grating scattering wavevectors and the photon propagation is optimised to achieve highly efficient surface THz emission via dual lobe beam profiles, symmetrically placed at 25° from the surface normal, a peak optical power of 240 mW, and the highest slope efficiency (570 mW/A at 78 K and 700 mW/A at 20 K) reported to date in an electrically pumped multimode, surface-emitting disordered THz laser. Switching between multimode and single-mode emission is achieved by adjusting the lithographic pattern, to engineer the resultant photonic pseudo-bandgaps. Furthermore, a frequency tuning of 20 GHz is demonstrated by coupling the laser to an external mirror, driven by a piezoelectric actuator.

The possibility of easily switching from single-frequency-mode to multimode emission, while maintaining high output powers and a large slope efficiency, clearly unveils that our photonic quasi-crystal provides a robust performance enhancement for both regimes, very differently from all previously reported architectures that instead operate either with a single laser mode or a broad bandwidth^24–39,43^.

## Results

### Device architecture

Our 1D photonic architecture exploits an array of open slits lithographically defined in the top metal surface of the laser. The array induces a local spatial modulation of the refractive index, which determines the photon scattering mechanism for light feedback and extraction. The distribution of the slits is generated through a deterministic algorithm implementing the Octonacci quasi-crystal sequence^45^^,46^ via a custom Matlab code. The mathematical definition of this quasi-periodic sequence *S*_n_ follows a deterministic generation rule based on two initial elements *S*_1_ = B and *S*_2_ = A, from which the *n*_th_-order sequence *S*_*n*_ = *S*_*n*-1_*S*_*n*-1_*S*_*n*-2_ is derived for *n* > 2. The lowest-order sequences are *S*_3_ = AAB, *S*_4_ = AABAABA and *S*_5_ = AABAABAAABAABAAAB. An alternative method to build the Octonacci series involves the application of the so-called substitution rule: A→B and B→BAB. For the *n*_th_-order Octonacci sequence, this rule implies defining the number *n*_A_ of A-elements and the number *n*_B_ of B-elements. The resulting total number of elements is given by the Pell number *P*_*n*_ = *n*_A_ + *n*_B_, defined as *P*_*n*_ = 2*P*_*n*−1_ + *P*_*n*−2_ for *n* > 2, with *P*_1_ = 0 and *P*_2_ = 2^46^.

**Fig. 1:**
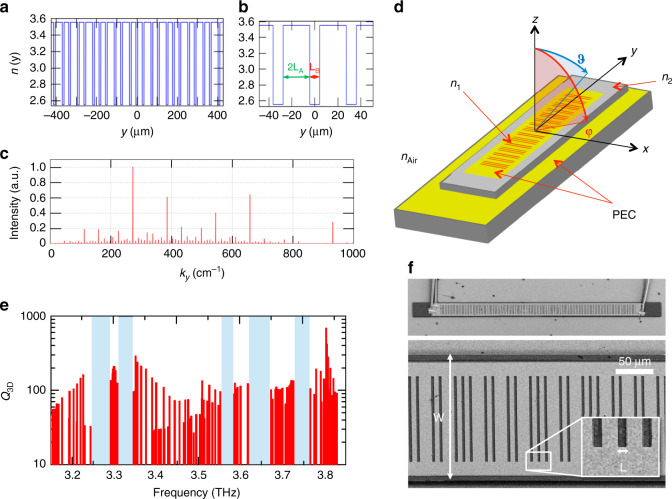
Device concept and simulation. **a** Spatial dependence of the effective refractive index induced by one-dimensional patterns of open slits defined following the 8th-order Octonacci sequence, with geometric parameters *L*_A_ = 11.6 µm and *L*_B_ = 8.7 µm that generate a quasi-periodicity of *Λ* = 2*L*_A_ + *L*_B_ = 31.9 µm. **b** Enlarged section of the refractive index spatial modulation induced by the Octonacci grating, showing the characteristic length *L*_B_ associated with the open slit and the length *L*_A_ associated with the metal-covered regions. **c** Fourier space representation of the grating wavevectors, highlighting the main Bragg peaks contributing to the photon scattering inside the photonic quasi-crystal laser. **d** Device schematics. A fully three-dimensional device simulation is performed through a finite element method (FEM) to extract the resonating modes via Maxwell’s equations. The laser active material is modelled with a refractive index *n*_1_ = 3.6, while the external border of the ridge covered by a 7-nm-thick Cr layer is described by the effective complex value *n*_2_ = 4.43 + i0.31, accounting for the optical losses induced by chromium. The laser is surrounded by a volume of air with *n*_Air_ = 1. The top and bottom metal claddings of the laser are described by perfect electric conductor (PEC) conditions. The far-field polar coordinates are defined as follows: *φ* is in the x-z plane, and *ϑ* is in the y-z plane. **e** Plot of the quality factor as a function of the resonance frequency calculated for a three-dimensional model of a device with a ridge width of *W* = 160 µm, slit length of *L* = 1.9 µm, and quasi-period of *Λ* = 31.9 µm. The light blue areas indicate the photonic pseudo-bandgap associated with the Octonacci quasi-crystal. **f** SEM image of a prototypical device with a ridge width of *W* = 160 µm and length of 2.9 mm, featuring the Octonacci grating on the laser top surface with a slit length of *L* = 3.5 µm

In the present work, we implement the Octonacci pattern on the top metal surface of a double-metal cavity using the following approach (see Methods). For each B-element of the Octonacci sequence, an air slit of length *L*_B_ is introduced into the top metal surface, while for each A-element, a metal-covered segment of length *L*_A_ is included (see Fig. 1b). The generated Octonacci quasi-crystal grating is characterized by a quasi-periodicity *Λ* = 2*L*_A_ + *L*_B_, which gives rise to a scattering wavevector *k*_0_ = 1/*Λ*. Transfer matrix studies of the one-dimensional Octonacci grating^45,46^ demonstrated that a number of photonic pseudo-bandgaps appear around the frequency *ν*_0_ = *ck*_0_/*n*_eff_, where *c* is the light speed in vacuum and *n*_eff_ is the effective material refractive index. These pseudo-photonic bandgaps are found within the approximate spectral interval 0.6*ν*_0_ – 1.4*ν*_0_. The edges of the pseudo-bandgaps are usually associated with the presence of electromagnetic modes with high quality factors and large radiative losses and are typically responsible for lasing.

Considering a two-dimensional effective index model^47^ for the case of a desired photon frequency of ~3.3 THz, the air slit can be described with an effective refractive index of *n*_B_ = 2.55, while in the metal-covered area, the effective refractive index is *n*_A_ = 3.55. We initially set the Octonacci lengths to *L*_B_ = 8.7 µm and *L*_A_ = 11.6 µm to define an 8th-order Octonacci grating that induces the spatial modulation of the refractive index shown in Fig. 1a. The corresponding spatial Fourier transform features a number of Bragg peaks (Fig. 1c) responsible for the surface extraction. These peaks, in the reciprocal space of the quasi-crystal, are exploited to achieve the scattering of the photons propagating in the resonator, which is necessary to provide light feedback and extraction. The quasi-periodicity is set to *Λ* = 31.9 µm so that the one-dimensional transfer matrix approach predicts multiple photonic bandgaps for TM-polarized radiation in the spectral range approximately between 2.0 THz and 4.5 THz. To perform a deeper investigation of the devised resonators, a finite element method (FEM) commercial solver is used to implement a fully three-dimensional electromagnetic model of the Octonacci double-metal waveguide. The GaAs/AlGaAs-based active region heterostructure^48^ is described with a refractive index *n*_1_ = 3.6, as shown in Fig. 1d. Since the ridge external border is covered by a 7-nm-thick Cr layer to suppress undesired modes, extending to the resonator edges, the Cr layer and the heterostructure underneath are modelled as an effective medium with a complex index of *n*_2_ = 4.43 + i0.31, accounting for the optical losses induced by chromium. This value has been retrieved from the simulation of the transverse propagation of a 3.5-THz mode in a 10-µm-thick GaAs slab covered by 7 nm of chromium, using the material constants indicated in Refs. ^49,50^.

The top and bottom metal claddings of the laser are described by exploiting perfect electric conductor (PEC) conditions, and the laser is surrounded by a finite volume of air with *n*_Air_ = 1. To mimic light propagation in free space, scattering boundary conditions (SBCs) are implemented at the outer surface of the air volume. The resonator geometry is designed with a fixed ridge length of 2.9 mm, including the top metal cladding with the 8th-order Octonacci grating and the Cr-covered pads at the edges of the ridge. A set of resonators with distinct ridge widths of *W* = 40 µm, 60 µm, 80 µm, and 160 µm has been devised to explore the effect of a change in the lateral device size on the resonating eigenmodes and their quality factors. Furthermore, we varied the slit length *L* from the initial value of *L* = *L*_B_ = 8.7 µm to *L* = 1.9 µm and *L* = 3.5 µm, to limit the vertical optical losses while ensuring the necessary scattering for the electromagnetic radiation. Simulations were performed to compute the three-dimensional quality factor *Q*_3D_, including the in-plane Ohmic losses and the radiative losses associated with the light out-coupling in the vertical direction (Ref. ^39^). The numerical results (Fig. 1e and Supplementary Figs. S1a, S1b and S2a–c and S3a–c) indicate that, by keeping the slit length *L* fixed and increasing *W*, there is a greater number of pseudo-bandgaps in the investigated frequency range, i.e., between 3.1 THz and 3.9 THz, and also the average mode quality factors *Q*_3D_ simultaneously become significantly larger, both in the case of multimode lasers (see Fig. S1a–b) and single-frequency lasers (see Fig. S2a–c). As an example, for a narrow ridge width (*W* = 40 µm), the simulated *Q*_3D_ values are, on average, smaller than 40 (see Supplementary Information and Supplementary Fig. S2a), while for *W* = 160 µm, a relevant number of eigenmodes exhibit *Q*_3D_ > 100 owing to the improved lateral confinement of the radiation, as shown in Fig. 1e and Fig. S2c for *L* = 1.9 µm. Therefore, a smaller *W* can support a limited number of lasing modes, while larger slit lengths are potentially able to sustain richer multimode emission.

A representative scanning electron microscopy (SEM) image of a fabricated device with *W* = 160 µm and *L* = 3.5 µm is shown in Fig. 1f.

### Experimental characterisation

**Fig. 2:**
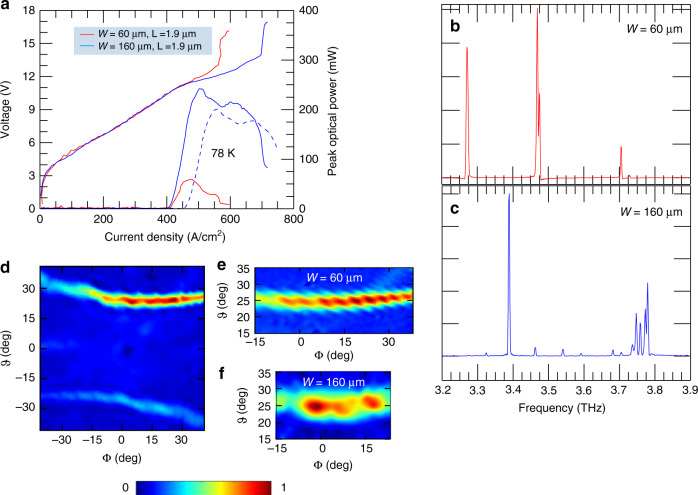
Multimode emitting devices. **a** Plot of the current density–voltage (J–V) and light–current density (L–J) characteristics of Octonacci lasers with a length of 2.9 mm and a slit length of *L* = 1.9 µm for different ridge widths of *W* = 60 µm and *W* = 160 µm. The measurements were performed at a heat sink temperature of 20 K, while driving the device in pulsed mode with a pulse width of 200 ns and a pulse repetition rate of 50 kHz (i.e., with a 1% duty cycle). The dashed blue curve represents the L–J characteristic of the device with *W* = 160 µm measured at 78 K, under identical driving conditions. The optical power scale was corrected to take into account the 75% transmission of the cryostat polyethylene window and the radiation collection efficiency of the pyroelectric detector. **b** Fourier transform infrared (FTIR) emission spectrum of the device with *W* = 60 µm and *L* = 1.9 µm operated at 12 K with a 4% duty cycle, acquired with a spectral resolution of 0.125 cm^−1^ **c** FTIR emission spectrum of an Octonacci laser with a ridge width of *W* = 160 µm and slit aperture length of *L* = 1.9 µm, operated at a heat sink temperature of 12 K and a 1% duty cycle. Up to 13 distinct modes are visible, with −20 dB mode suppression. **d** Far-field intensity pattern of an Octonacci laser with *W* = 60 µm and *L* = 1.9 µm, measured by scanning the pyroelectric detector on a spherical surface at a distance of 7 cm from the laser top plane. The laser was driven in pulsed mode (pulse frequency of 50 kHz and pulse width of 200 ns) at a heat sink temperature of 20 K and at *J* = 500 A/cm^2^. The far-field angles (*φ*, *ϑ*) are defined according to the scheme in Fig. 1d. **e** Plot of the most intense far-field emission lobes for the device with *W* = 60 µm (also included in Fig. 2d) and **f** the brightest lobe for the laser with *W* = 160 µm, featuring the same slit aperture length of *L* = 1.9 µm. Both devices were operated under peak emission conditions with a 1% duty cycle

Based on the aforementioned simulations, a set of devices with different ridge widths *W* and slit sizes *L* were fabricated, as detailed in the Methods section, to evaluate how these geometrical parameters affect the laser performance. Figure 2a shows the experimental current density–voltage (J–V) and current density-peak optical power (J–L) characteristics measured for resonators with the same surface patterning (slit length *L* = 1.9 µm) but different ridge widths *W* = 60 µm and 160 µm. At a heat sink temperature of 20 K, the peak optical power reaches a maximum value of 240 mW for the device with *W* = 160 µm, corresponding to a slope efficiency of SE ≈ 720 mW/A and a wall-plug efficiency of *η* ≈ 1%. By reducing the ridge width to *W* = 60 µm, the peak optical power is consequently reduced (≈57 mW), with SE ≈ 420 mW/A and *η* ≈ 0.5%, due to the smaller available active region volume for laser amplification and the reduced extraction efficiency owing to the smaller slit area. The threshold current densities of the devices are almost the same, with 412A/cm^2^ and 420 A/cm^2^ for *W* = 160 µm and *W* = 60 µm, respectively. Remarkably, the power output for the larger ridge width is still 200 mW at 78 K with a corresponding slope efficiency of ≈570 mW/A.

The Fourier transform infrared (FTIR) emission spectrum of the device with *W* = 60 µm and *L* = 1.9 µm shows five optical modes (Fig. 2b); the main modes are located at 3.280 THz, 3.480 THz and 3.485 THz, respectively, with some less intense lines at ~3.72 THz, covering a total emission of 440 GHz. Visibly larger multimode emission occurs when the ridge width is significantly increased while keeping the slit length fixed. The resulting FTIR emission spectrum (Fig. 2c), collected while keeping the operating conditions fixed, shows a dominant optical mode at 3.39 THz with 12 other spectral lines between 3.30 THz and 3.78 THz, over a 530-GHz-wide spectral interval (including the small minor peaks located at 3.28 THz and 3.81 THz). This result is in agreement with the numerical prediction that a much broader frequency bandwidth is supported by devices with a larger W. A comparison of the experimental data and simulations shows that, in both cases, the more intense peak is very close to the upper edge of a photonic pseudo-bandgap centred at ~3.35 THz, while the minor peaks between 3.7 THz and 3.8 THz correlate well with the high-quality-factor optical modes located within a frequency bandwidth centred at 3.75 THz.

The corresponding far-field intensity profiles were measured by scanning a pyroelectric detector, mounted on a motorised stage, across a spherical surface. Figure 2d shows the far-field radiation distribution of an Octonacci laser with *W* = 60 µm and *L* = 1.9 µm driven in a pulsed regime with a 1%-duty cycle at a temperature of 20 K and at the maximum peak optical power. The surface-emitted radiation is concentrated in two main lobes elongated along the horizontal direction, at elevation angles of approximately *ϑ* ≈ −25° and *ϑ* ≈ +25°. The most intense lobe is concentrated within an angular span of Δ*ϑ* ≈ 5° and Δ*φ* ≈ 60°. To compare the far-field profiles of lasers with different values of *W*, we increased the spatial resolution when scanning around the most intense lobe. A comparison of the intensity distribution patterns of two devices with the same Octonacci patterns and different ridge widths, *W* = 60 µm (Fig. 2e) and *W* = 160 µm (Fig. 2f), shows that emission occurs along the same elevation angle *ϑ* ≈ +25° with a vertical divergence in the 5°–8° angle, as expected in the case of photonic structures with the same grating distribution. The vertical divergence is slightly narrower for *W* = 60 μm (Δ*ϑ* ≈ 5°) than for *W* = 160 μm (Δ*ϑ* ≈ 8°), which is most likely an effect induced by the different sizes of the resonators.

However, the horizontal divergence becomes narrower (Δ*φ* ≈ 40°) when the ridge width is increased, which in turn allows a longer slit transverse size, limiting diffraction effects. The far-field pattern distribution is the result of the near-field electromagnetic distribution, governed by the complex nonlinear mixing and interactions of the multiple wavevectors associated with the grating Bragg resonances (Fig. 1c) and the photon wavevector, in combination with nonlinear effects in the active material.

**Fig. 3:**
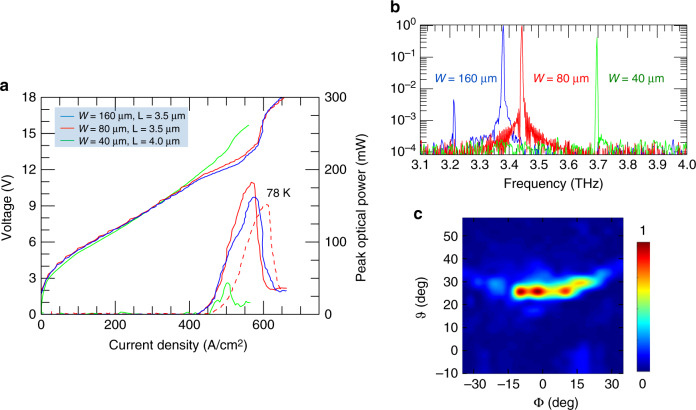
Single-frequency-mode emitting devices. **a** V–J and L–J characteristics of a set of 2.9-mm-long Octonacci lasers with ridge widths of *W* = 160 µm, *W* = 80 µm and *W* = 40 µm. The slit length ranges between *L* = 3.5 µm (for *W* = 80–160 µm) and *L* = 4.0 µm (for *W* = 40 µm). The L–J–V characteristics were measured at a heat sink temperature of 20 K in pulsed mode with a 1% duty cycle (pulsed width of 200 ns and a pulse repetition rate of 50 kHz). The dashed blue curve represents the L–J characteristic of the device with *W* = 80 µm, measured at 78 K, under identical driving conditions. The optical power scale was corrected to take into account the radiation collection efficiency of the pyroelectric detector and the 75% transmission of the polyethylene window of the cryostat**. b** FTIR emission spectra of the single-frequency-mode devices with *W* = 160 µm (blue), *W* = 80 µm (red) and *W* = 40 µm (green) operated at 12 K in pulsed mode with a duty cycle of 4%, measured with a resolution of 0.125 cm^−1^ and averaging over 32 scans. **c** Far-field intensity patterns measured 7 cm away from the top surface of a laser with *W* = 160 µm and *L* = 3.5 µm, while keeping the heat sink temperature fixed at 15 K with a duty cycle of 1%, at *J* = 550 A/cm^2^

We then fabricated a set of devices exploiting the same 8th-order Octonacci grating, but with larger slit sizes, to investigate how light scattering and extraction are influenced by the slit geometry/dimensions and if this design concept can also allow single-frequency-mode emission. Lasers with the same slit length *L* = 3.5 µm and ridge widths of *W* = 80 µm and 160 µm show comparable peak output powers of ≈190 mW and ≈160 mW, respectively (Fig. 3a). By reducing the ridge width to *W* = 40 µm and using a slit length of *L* = 4.0 µm, the optical power decreases to ≈40 mW as a consequence of the reduced volume of the active material. The most powerful device (with *W* = 80 µm and *L* = 3.5 µm) is single-frequency-mode and displays a slope efficiency of ~700 mW/A (570 mW/A at 78 K) and a wall-plug efficiency of 1% (0.8% at 78 K), the current record for double-metal THz QCL resonators^51^.

The corresponding FTIR emission spectra (Fig. 3b) show robust single-mode emission with a visible red shift from 3.7 THz to 3.38 THz as *W* is increased across the 40 μm–160 µm range. Interestingly, the observed ridge-width-dependent shift is in good agreement with the results of the FEM simulations. It is worth mentioning that for the device with the largest ridge width (*W* = 160 μm), a second, very weak, spectral line is visible at 3.22 THz with an intensity that is ≈−20 dB lower than that of the main peak at 3.38 THz. This result indicates that excessively large lateral sizes of the ridge are inefficient in suppressing high-order lateral modes with the same size/thickness of the absorbing boundary.

The far-field intensity pattern of one of the most powerful lasers is shown in Fig. 3c. The single-lobe beam shape, which is 90% more intense than the second symmetric lobe, is consistent with the results for multimode surface-emitting lasers and shows a main radiation spot concentrated at an angle of *ϑ* ≈ +25° with a divergence of Δ*ϑ* ≈ 5° and Δ*φ* ≈ 35°, which is slightly narrower than that of the multimode devices (Fig. 2d, 2e). Δ*ϑ* ≈ 5° indicates a beam that is ~2.4 wider than the diffraction-limited divergence, which implies operation in approximately three spatial modes.

The comparison between the fabricated set of devices indicates that lasers with smaller slit lengths (*L* = 1.9 µm) feature rich multimode emission, while larger slits induce a much narrower spectral bandwidth, irrespective of the device width W. To investigate this effect further, numerical simulations of the three-dimensional quality factors of the resonators were performed and compared to the experimental FTIR spectra (see the Supplementary Information file). Figure S1b shows a scatter plot (top) with the relative mode suppression (MS), defined as MS = 10 log_10_ (*I*_min_/*I*_max_), of the experimental spectral peaks, which are in relatively good agreement with the band edges of the computed photonic pseudo-bandgap (bottom) for an Octonacci resonator with a ridge width of *W* = 160 µm and a slit length of *L* = 1.9 µm. The comparison between the MS factor and the quality factors of the optical modes in the investigated frequency bandwidth for a resonator with the same ridge width but a larger slit aperture (*L* = 3.5 µm) (Fig. S2c) highlights the presence of multiple, narrower pseudo-bandgaps in correspondence with the two experimental spectral lines. As shown in Fig. S3, the grating aperture length also affects the radiative efficiency, which is defined as the ratio between the radiative decay frequency of the mode, which is obtained from the integral of the Poynting vector all around the resonator, divided by the amount of electromagnetic energy stored inside the resonator, and the imaginary part of the complex eigenfrequency, accounting for all decay channels.

Indeed, for the same ridge width of *W* = 160 µm (Fig. S1b), the computed photonic pseudo-bandgaps for *L* = 1.9 µm are red-shifted by a few tens of GHz with respect to a device exploiting slit apertures with *L* = 3.5 µm (Fig. S2c). The radiative efficiency (S3a and zooms in the panels S3b and S3c) associated with the lowest band-edge modes is higher in the case of *L* = 3.5 µm, as a consequence of the larger extraction area provided by the quasi-crystal apertures. The spectral peaks measured at 3.22 THz and 3.38 THz for this Octonacci laser (upper panel Fig. S2c) match well with the computed lower-band-edge modes (bottom panel in Fig. S2c), which indeed exhibit a larger radiative efficiency than their associated upper-band-edge modes.

To further control the emission of the Octonacci resonators, we also performed a coupled-cavity experiment to finely tune the emitted spectral lines. The resonators were coupled to an external gold-coated mirror, placed parallel to the top metal surface at a distance *d*, which was controlled via a piezo-driver to scan a range *d* = 80–430 μm in the z-direction (where 0 μm is the top surface of the laser). As a consequence, a double-metal cavity is formed. By sweeping the mirror position, the external cavity creates resonances at frequencies *ν*_cav_ = (*mc*)/(2*d*), where *c* is the speed of light in vacuum and *m* is the order of the resonance. Therefore, the different lasing modes are brought in and out of resonance with the bare external cavity modes *ν*_cav_. Equivalently, for light emitted at a wavelength *λ*, the reflection from the external mirror can induce frequency and intensity modulation of the spectral lines due to resonant coupling when *d* approaches *λ*/2, *λ*, 3*λ*/2, 2*λ*, etc.^52,53^. Based on coupled mode theory, complex spectral dynamics are expected for a multimode, surface-emitting device, which could exchange energy via a cross-coupling interaction with the electromagnetic modes of the external cavity. Moreover, inside the Octonacci resonator, the different modes may interact, due to mirror-induced self-coupling^54^.

**Fig. 4:**
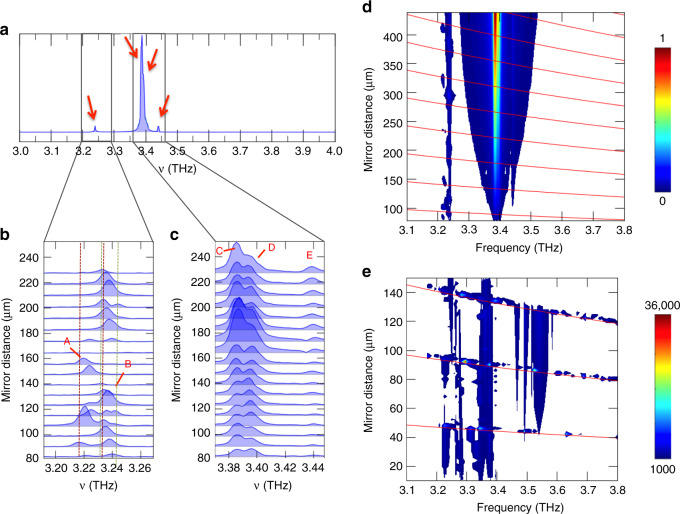
External cavity tuning. **a** FTIR emission spectrum of the 2.9-mm-long Octonacci laser (*W* = 160 µm and *L* = 1.9 µm), operated at a temperature of 12 K and a 1% duty cycle, emitting multiple spectral lines labelled as: mode A (3.243 THz), mode B (3.235 THz), mode C (3.386 THz), mode D (3.396 THz), and mode E (3.440 THz). The emitted light is collected while moving a gold mirror positioned parallel to the laser top surface at a distance *d*, which is controlled by a piezoelectric driver. **b** Spectral dynamics of the emission for modes A and B at different mirror positions in the range 80 μm–230 µm. The dashed vertical lines indicate the tuning range of mode A (dashed brown lines) and mode B (dashed green lines). **c** Evolution of modes C, D and E as the mirror position is swept between 80 µm and 230 µm. **d** Contour plot of the emission spectra acquired by sweeping the mirror between 80 µm and 430 µm, highlighting the strong frequency dynamics at ~3.25 THz (modes A and B). The spectral lines at ~3.39 THz (modes C, D and E) are less influenced by the mirror movement. Each spectrum is acquired with a mirror position shift of 18 µm, and the intensity of the spectral lines is normalized, as described by the lateral colour bar. The red lines show the external cavity (comprising the top laser surface and the external mirror) frequency *ν*_cav_ as a function of the mirror position *d*. **e** Contour plot of the quality factor of the simulated resonating modes for the device with *W* = 160 µm and *L* = 1.9 µm in the presence of a mirror at a distance *d* in the 10 μm–150 µm range. When the bare external cavity frequencies (red lines) match the laser eigenmode frequencies at the appropriate mirror positions, the overall three-dimensional quality factor is strongly enhanced, as a result of the resonant coupling at 3.25 THz and 3.39 THz, in good agreement with the experimental results. A lower cut-off has been artificially set in panels d and e, so that the white areas represent the noisy spectral regions, i.e., the spectral windows without THz emission

Figure 4a–c show the FTIR emission spectrum of an Octonacci laser with a ridge width of *W* = 160 µm and a slit length of *L* = 1.9 µm. Five main modes can be identified over the entire mirror dynamic range: mode A (3.243 THz), mode B (3.235 THz), mode C (3.386 THz), mode D (3.396 THz) and mode E (3.440 THz). Figure 4b shows the spectral dynamics and the mode hopping of modes A and B, while Fig. 4c highlights the evolution of lines C, D and E as a function of the mirror distance. In both sets of spectral lines, a modulation of both the relative peak intensities and the frequency is observed. By scanning the mirror over the whole 80 μm–430 μm distance (contour plot in Fig. 4d), the lower-frequency modes A and B exhibit mode hopping with a maximum frequency shift of ~20 GHz, much larger than the shift recorded for modes C, D and E, which show a maximum tuning of ≈6 GHz for peak E, in the best case. The dynamics of modes A and B in Fig. 4b reveal a mode-hopping effect, in combination with the appearance of a third mode for a mirror distance of 115 μm. These effects are induced by the interplay of cross-coupling and self-coupling interactions, which become dominant for very short mirror distances below 150 μm.

Figure 4c indicates that peaks C and D are less influenced by the mirror distance variation. The relative height of these two peaks indeed shows an overall change of ≈±30%, and the overall intensities of modes C and D tend to decrease for smaller mirror distances. This trend is a consequence of the decreased lateral leakage of radiation when the external microcavity thickness becomes very small, so that a smaller fraction of light is funnelled to the FTIR sensor.

We then compare these experimental results with simulations of the three-dimensional quality factors of the eigenmodes of a system comprising the Octonacci laser and a parallel mirror placed at a fixed distance *d* from the top laser surface. The contour plot (Fig. 4e) of the quality factor of the resonating modes, simulated for the device with *W* = 160 µm and *L* = 1.9 µm, at mirror distances of 10 µm < *d* < 150 µm shows that the three-dimensional quality factor is strongly enhanced when the mirror is positioned, so that the bare external cavity frequency is resonant with a laser eigenmode frequency; therefore, the results show good agreement with the experimental coupling retrieved for the spectral peaks located at 3.25 THz and 3.39 THz. It is worth mentioning that the experimental mode dynamics are expected to be even more complex here due to the interplay between the nonlinear cross-coupling and self-coupling interactions among different cavity modes, which has not been included in our numerical model. This can easily lead to amplitude modulation effects between the emitted optical modes, as observed in the present experiment.

Finally, to prove that the scattering mechanism induced by the selected Octonacci photonic pattern is able to strongly enhance the light extraction from the laser active region, leading to major enhancements of the overall device performance, independent of the selected active region/MBE growth quality, we summarize, in Table 1, the comparison between the peak optical power and the slope efficiency of a set of 1D and 2D photonic designs, fabricated from the same active region heterostructure. The related current–voltage and light–current characteristics are reported in the Supplementary Information (Fig. S4). The comparison clearly unveils that, in the case of multimode disordered resonators with identical dimensions, the selected Octonacci pattern leads to a 40 time increase in the slope efficiency with respect to random^39^ lasers and a 11 time increase with respect to the quasi-crystal^44^ lasers, with a major increase in the emitted power. Additionally, alternative single-frequency-mode 1D DFB emitters^27,28^, relying on a different concept for feedback/extraction, exhibit a power decrease of up to more than an order of magnitude and a related significant reduction in the slope efficiency, clearly highlighting the superior quantum efficiency of the quasi-crystal, tunable lasers demonstrated in the present work.

## Discussion

In conclusion, we have demonstrated highly efficient surface-emitting semiconductor lasers operating at THz frequencies, exploiting quasi-crystalline distributed feedback photonic patterns. The architecture is based on a slit grating in the laser top metal surface following the Octonacci sequence pattern, which controls and determines the light feedback and extraction mechanisms in the cavity. By tuning the ridge width and the slit aperture length, the light extraction is carefully controlled to achieve multimode or single-frequency-mode emission with maximum peak optical powers of 240 mW (190 mW in single-frequency-mode operation), corresponding slope efficiencies of ≈720–700 mW/A (570 mW/A at 78 K) and a maximum wall-plug efficiency of *η* ≈ 1%, together with low-divergence, double-lobed emission patterns. Independent of the device width, the resonators with smaller aperture lengths (*L* = 1.9 µm) feature rich multimode emission spectra over a maximum frequency bandwidth of 530 GHz, in good agreement with the numerical simulations. Octonacci lasers with larger slit lengths emit in single-frequency-mode operation within the spectral gain bandwidth of the active material. Frequency tuning of the laser line up to 20 GHz for specific resonating modes is demonstrated. Finally, it is worth mentioning that, once compared with THz QCL VECSELs^55,56^, our Octonacci lasers provide a very efficient technological solution to achieve a proper combination of a large bandwidth and a high peak pulse power; however, these characteristics are achieved at the expense of more divergent optical beams, resulting in an inherently low spatial coherence.

The capability to flexibly tune the radiation loss via the use of quasi-crystal photonic heterostructures provides a valuable route for multicolour imaging, spectroscopic and metrological applications, while ensuring high spectral purity of the emission lines.

## Materials and methods

### Growth and fabrication

The GaAs/Al_0.15_Ga_0.85_As QCL heterostructure was grown by molecular beam epitaxy on an undoped GaAs substrate. The active region features a three-quantum-well architecture with a single extractor well^44^. The layer sequence is **5.5**/11.0/**1.8**/11.5/**3.8**/9.4/**4.2**/18.4 (in nm), where the Al_0.15_Ga_0.85_As layers are shown in bold face, GaAs is shown in roman font, and the underlined number indicates a Si-doped layer with a density of 2 × 10^16^ cm^−3^. The active region growth is terminated by a 700-nm-thick highly doped (2 × 10^16^ cm^−3^) GaAs contact layer, with an Al_0.5_Ga_0.5_As etch-stop layer on the top. After growth, Au–Au thermo-compressive wafer bonding of the QCL wafer onto an n^+^-GaAs carrier wafer was performed. After selective wet etching of the host GaAs substrate and the Al_0.5_Ga_0.5_As etch-stop layer, the active region was coated with a top metal layer of Cr/Au (5 nm/150 nm). By using optical lithography, the sample surface was patterned with air slits, whose centre positions correspond to the Octonacci sequence realised with a MATLAB code. The resonators were fabricated by varying the slit length *L* and the laser ridge width *W*. The 700-nm-thick n+ top contact layer was completely removed below the etched slits by means of an inductively coupled reactive-ion etching (ICP-RIE) process; as a result, the cavity losses were reduced, and the light extraction was optimised. An external 7-nm-thick Cr border was fabricated on the active region using optical lithography, surrounding the gold top metal region and implementing strongly absorbing boundary conditions. Under the Cr border, the n^+^ top contact layer was not etched away to enhance the suppression of modes extending towards the edge of the resonator. A final ICP-RIE process was required to etch down the ridge with vertical sidewalls. Finally, individual devices were cleaved and indium-soldered onto a copper sub-mount and wire-bonded at the two edges of the long side of the ridge to minimise the perturbation of the far-field emission profile by the gold wires.

## Supplementary information

Supplementary Information file
