# Supplementary material for: Highly efficient surface-emitting semiconductor lasers exploiting quasi-crystalline distributed feedback photonic patterns
Source: Light Sci Appl. 2020 Apr 9;9:54. doi: 10.1038/s41377-020-0294-z (PMC7142150; doi:10.1038/s41377-020-0294-z)
Supplement: Supplementary file 1 — Supplementary Information file [file 41377_2020_294_MOESM1_ESM.docx]

**Supplementary Information**

**Highly efficient surface-emitting semiconductor lasers exploiting quasi-crystalline distributed feedback photonic patterns**

**Simone Biasco,^1*^ Andrea Ciavatti,^1*^ Lianhe Li,^2^ A. Giles Davies,^2^ Edmund H. Linfield,^2^ Harvey Beere,^3^ David Ritchie,^3^ and Miriam S. Vitiello^1^**

*^1^NEST, CNR - Istituto Nanoscienze and Scuola Normale Superiore, Piazza San Silvestro 12, 56127, Pisa, Italy*

*^2^School of Electronic and Electrical Engineering, University of Leeds, Leeds LS2 9JT, UK*

*^3^Cavendish Laboratory, University of Cambridge, Cambridge CB3 0HE, UK*

* these authors, listed in alphabetic order, contributed equally to this work

**1. Evolution of the photonic pseudo-bandgaps with the laser ridge width W**

The implemented electromagnetic simulations of the Octonacci resonators exploit the finite element method (FEM) to retrieve the resonating eigenmodes in the device, in the framework of a fully three-dimensional vector model.

The computed three-dimensional quality factor Q_3D_ plot show that there are ranges of frequencies were no modes are allowed, forming the so-called photonic pseudo-bandgap for aperiodic systems. This influences the emission frequencies of the lasers, since the modes at the edges of the photonic bandgaps often have high quality factors and are favored for lasing. The distribution of the bandgaps in the spectral range of interest, i.e. the active material gain bandwidth, and the frequency width of these forbidden spectral regions are strongly dependent on the geometric parameters of the devices, such as the slit size L and the ridge width W.

Figure S1a reports the computed Q_3D_ for an Octonacci THz laser having L = 1.9 µm and ridge width W = 60 µm, unveiling the presence of multiple photonic pseudo-bandgaps (red shaded intervals) at frequencies where no modes can resonate in the device. In particular, the interval between 3.25 THz and 3.44 THz features band-edge modes with relatively high quality factors of about 80 for the lower band edge and about 300 for the upper band edge. Instead a limited number of modes with Q_3D_ ≈ 25 lies in this spectral region. Much narrower photonic bandgaps are present for frequencies larger than 3.5 THz, with typical width of 20 GHz - 25 GHz, as highlighted by the red shades.

Comparing this results with that achieved on the laser bar having W = 160 µm (see Fig.1e and S1b), it comes out that, the latter supports a larger number of modes with Q_3D_ > 100, all located around 3.3 THz. Two distinct bandgaps (blue shaded intervals) appear in the intervals 3.24 THz-3.28 THz and 3.31 THz-3.34 THz. For frequencies larger than 3.5 THz, multiple large pseudo-bandgaps are present, with a frequency width ranging from 30 GHz to 55 GHz approximately.

**Figure S1. Quality factor and corresponding photonic band gap. (a)** Plot of the quality factor as a function of the resonating frequency calculated for a three dimensional model of a device with ridge width W = 60 µm, slit length L = 1.9 µm. The light red areas indicate the photonic pseudo-bandgap associated with the Octonacci quasi crystal. The upper panels show the relative mode suppression (MS) of the experimental spectral peaks, directly compared to the calculated photonic pseudo-bandgap. **(b)** Three-dimensional FEM simulation of the quality factor of the eigenmodes for a 2.9 mm-long Octonacci laser with W = 160 µm and L = 1.9 µm. The upper panel shows the relative mode suppression (MS) of the experimental spectral peaks, directly compared to the calculated photonic pseudo-bandgap. The rich multimode emission is characterized by a relatively low mode suppression with a maximum of -17 dB.

**2. Comparison between simulation ad experimental emission spectra**

Figures S1 and S2 report a series of plots in which the simulated quality factors Q_3D_ and photonic pseudo-bandgaps are directly compared to the experimental spectral peaks of different Octonacci lasers measured with FTIR spectroscopy. The j-th mode with intensity I_j_ is indicated in a scatter plot showing the relative mode suppression (MS), defined as MS =10 log_10_ (I_j_/I_max_), where I_max_ is the intensity of the highest peak in the spectrum.

Figure S1a reports the MS and Q_3D_ for the multimode Octonacci device with W = 60 µm and L = 1.9 µm, which highlights the correspondence of the strongest spectral lines at ≈ 3.27 THz and 3.46 THz with the numerical prediction of the band edge frequencies. Instead, the less intense experimental lines at ≈ 3.7 THz may be associated with the narrower pseudo-bandgaps supported by the photonic structure around this frequency. If one compares these data with the ones retrieved on the larger laser ridge, having the same L value (W = 160 µm, Figure S2a) is appears that, in the case of wider cavities, most experimental peaks are in good correspondence with high-quality-factor eigenmodes computed in the simulation, even if the spectral lines between 3.7 THz and 3.8 THz actually lie in a photonic pseudo-bandgap. This can be explained with the limitations and assumptions of the model, which neglects non-linear effects in the active material, such as the gain mode competition.

**Figure S2. Quality factors. (a-c)** Results of the three-dimensional FEM simulation of the quality factor of the eigenmodes for a 2.9-mm-long Octonacci laser with W = 40 µm and L = 4 µm (a); with W = 60 µm and L = 3.5 µm (b); with W = 160 µm and L = 3.5 µm (c). The upper panel shows the relative mode suppression (MS) of the experimental spectral peaks, directly compared to the calculated photonic pseudo-bandgap

Figure S2a shows the trivial MS = 1 for a single-mode Octonacci device emitting at 3.7 THz, having W = 40 µm and larger slit size L = 3.5 µm. This emission spectrum is in very good agreement with the FEM simulation, which indicates there are eigenmodes with Q_3D_ > 100 around this frequency, emerging from a background of modes having an average Q_3D_ < 40, due to the poor confinement associated to the very narrow resonator. These data can be also compared with those achieved when the ridge width is increased (W = 80 µm, L = 3.5 µm, Figure S2a) (W = 160 µm, L = 3.5 µm, Figure S2c). In this latter two case, the most intense spectral peak is in good qualitative correspondence with the bandgap centered at 3.43 THz (for W=80 µm) and 3.40 THz (for W=160 µm), respectively, which features band-edge modes with high quality factors. The second very weak spectral line in the case of W = 160 µm, L = 3.5 µm, (having MS > -20 dB) is instead associated with the computed lower-band edge modes located below 3.25 THz.

The presented analysis show that there is a good qualitative agreement between the measured emission spectra and the computed photonic pseudo-bandgaps of the Octonacci lasers having different geometric parameters.

**3. Radiative efficiency**

**Figure S3. Radiative efficiency. (a)** Radiative efficiency calculated from a three-dimensional simulation of the resonating modes in an Octonacci laser with L = 1.9 µm (red) and L = 3.5 µm (blue), and fixed width W = 160 µm. The computed photonic pseudo-bandgaps for L = 1.9 µm are red-shifted by few tens of GHz with respect to those for L = 3.5 µm. (b-c) zoomed windows of panel (a).

**4. Current-voltage and light-current characteristics of a set of 1D and 2D QCL resonators.**

Figure S4 shows the current-voltage (I-V) and light-current (L-I) characteristics of a set of 1D (3^rd^ order DFB and corrugated wire laser) and 2D (random laser and 7-fold quasi crystal laser) photonic designs we fabricated from the same active region heterostructure. The comparison clearly shows that, despite the threshold current density remains practically unperturbed the Octonacci quasi-crystal lasers provide a significantly larger power output.

**Figure S4. I-V and L-I characteristics of a set of 1D and 2D QCLs. (a)** I-V and L-I of a random laser having area 0.70 mm^2^, filling fraction 13%, and hole radius r = 8 µm (from Ref. 39), measured at a heat-sink temperature of 15 K while driving the lasers in pulsed mode with a pulse width of 200 ns and a repetition rate of 50 kHz (i.e. a 1%-duty cycle) in a nitrogen-purged environment. **(b)** I-V and L-I of a quasi-crystal THz laser (Ref. 45), having quasi-crystal periodicity *a* = 32.5 μm, filling factor 26.8% and area 0.7 mm^2^, measured at a heat-sink temperature of 15 K while driving the lasers in pulsed mode with a pulse width of 200 ns and a repetition rate of 50 kHz (i.e. a 1%-duty cycle) in a nitrogen-purged environment. **(c)** I-V and L-I of a corrugated wire laser having an area of 0.074 mm^2^ driven in pulsed mode with a pulse width of 200 ns and pulse repetition rate of 100 kHz, in a nitrogen-purged atmosphere. **(d)** I-V and L-I of a corrugated wire laser having an area of 0.074 mm^2^ driven in pulsed mode with a pulse width of 200 ns and pulse repetition rate of 100 kHz, in a nitrogen-purged atmosphere. In all cases optical power scales were corrected to account for the detector collection efficiency (integrating the measured optical power over the corresponding three-dimensional far-field intensity pattern) and the absorption of the polyethylene cryostat window (75%).

**Table S1. Performance comparison of a selection of aperiodic resonators.** Comparison between the peak output power and the slope efficiency of a set of 1D and 2D aperiodic THz lasers reported in the literature, fabricated from different active region heterostructures.
